# Pyuria in hospitalized general medical patients without urinary tract infection

**DOI:** 10.12688/f1000research.144853.2

**Published:** 2024-05-22

**Authors:** Praveenkumar Thyagaraju, Jharna Mandal, Hariswar Pari Thenmozhi, Surendran Deepanjali

**Affiliations:** 1 Medicine, Jawaharlal Institute of Postgraduate Medical Education and Research, Dhanvantri Nagar, Puducherry, 605006, India; 2 Microbiology, Jawaharlal Institute of Postgraduate Medical Education and Reserach, Dhanvantri Nagar, Puducherry, 605006, India

**Keywords:** microscopic pyuria, urinalysis, urinary tract infection, asymptomatic bacteriuria, general medical patients

## Abstract

Urine microscopy for detecting pus cells is a common investigation ordered in hospitalized general medical patients as part of routine care. A few previous studies have shown that sterile pyuria is not uncommon in this population. We studied the prevalence of pyuria among patients hospitalized with non-urinary tract infection (UTI) diagnosis in the medical wards. We excluded patients with asymptomatic bacteriuria (ASB). Pyuria was quantified in uncentrifuged urine using the chamber counting method, and ≥ 10 pus cells per mm ^3^ was considered significant. We also compared this method with the commonly used but less accurate method of counting pus cells/high power field using centrifuged urine (routine method). We studied 196 patients; 113 (57.7%) were males. Most (175[89.3%]) patients were hospitalized for an infection. We found that 18.4% of the study group had sterile pyuria, and it was strongly associated with the presence of concomitant microscopic hematuria (unadjusted odds ratio, 3.74 [1.65 to 8.50]; P=0.002). We found no association of pyuria with female gender, diabetes, acute kidney injury, or current antibiotic use. By routine method, 56 (28.6 %) patients had significant pyuria. In comparison to the chamber counting method, the routine method was 69.4(63—75.8) % sensitive and 80.6(75.1—86.2) % specific. The positive and negative predictive values were 44.6 (37.7— 51.6) % and 92.1 (88.4 — 95.9) %. We concluded that sterile pyuria and microscopic hematuria could be present in a proportion of hospitalized general medical patients without UTI or ASB. Clinical judgment is essential in interpreting the significance of abnormal urinalysis reports.

## Introduction

Urinalysis for detecting pyuria is a commonly ordered test in hospitalized patients. It is considered one of the ‘routine’ or ‘default’ investigations after hospital admission. In a retrospective cohort study using a national dataset of inpatient admissions from 263 hospitals in the United States from 2009 to 2014, Horstman et al. found that urinalysis was ordered in 47% of admissions. ^1^ They also found that the test is frequently repeated during hospital stay suggesting large-scale overuse. More recent studies show that urinalysis is often ordered without proper indication in the emergency department. ^2^^,^^3^

In patients with classic clinical symptoms and signs of UTI, the presence of pyuria and significant bacteriuria helps in ascertaining the diagnosis of UTI. Also, it has been observed that urinalysis serves as a useful “rule-out” test for UTI. ^4^ However, in those without typical clinical features of UTI, the presence of pus cells in urine detected by a routinely done microscopy examination often leads to diagnostic confusion. This is especially so if pyuria is accompanied by bacteriuria, which is detected by a urine culture. This condition, which was proposed to be called ‘Bacteriuria/Pyuria of Clinically Undetermined Significance (BPCUS),’ is indeed not so uncommon in hospital settings. ^5^

In a previous study including hospitalized general medical patients done in our centre, almost 80% patients who had asymptomatic bacteriuria (ASB) also had pyuria. ^6^ Even in the absence of a positive urine culture, a significant proportion of hospitalized patients could have pyuria, often referred to as ‘sterile pyuria.’ In a retrospective study done including 210 consecutive patients (both adults and children) with infections outside of the urinary tract and who were admitted under medical and surgical services of an academic medical center in Oklahoma, nearly one-third had >5 white blood cells (WBCs) per high-power field (hpf) of urine. ^7^

Pyuria can occur in non-infectious conditions like interstitial nephritis, diabetic nephropathy, and malignant hypertension also. ^8^ With this background, we studied the prevalence of significant microscopic pyuria among patients hospitalized with non-UTI diagnosis. We defined sterile pyuria as ≥ 10 WBCs per mm ^3^ of uncentrifuged urine using the chamber counting method, which is a more accurate method of quantifying pyuria. ^9^ We also compared this with the commonly practiced method of reporting the number of WBCs per high power field (hpf) in centrifuged urine sample (referred to as ‘routine method’ hereafter).

## Methods

We did a prospective observational study between April 2021 to June 2022 among adult patients presenting to casualty and who were subsequently admitted under the Department of Medicine, JIPMER. The study protocol was reviewed and approved by the Institute Ethics Committee (Human Studies), JIPMER, Puducherry (No. JIP/IEC/2020/202; dated April 19, 2021). Written informed consent was obtained from all study participants and the study was conducted in compliance with the Declaration of Helsinki. Our study population included adult patients hospitalized with a non-UTI diagnosis. We excluded patients who had a positive UTI symptom screening at admission as well as those on indwelling urinary catheter. We also excluded who had a recent (past 2 weeks) hospitalization, exposure to antibiotics or urinary catheterization. Patients were recruited within 24 hours of hospitalization. We defined positive UTI as the presence of at least two or more typical symptoms or signs suggestive of UTI like dysuria, urinary frequency, urgency, hesitancy, lower abdominal pain, loin pain, suprapubic tenderness, and renal angle tenderness.

Once the patients were recruited in the study, one of the investigators (TP) collected the clinical details from the patients. Patients submitted a fresh mid-stream urine sample (about 50 mL) in a sterile vial. A portion of this sample was further divided between 2 more sterile vials. One was sent to microbiology lab for culture. The second one was to the medicine laboratory, where a portion of the sample was subjected to dipstick analysis of proteinuria and glucosuria. The other portion was centrifuged at a rate of 1500 to 2000 rpm for 3 to 5 minutes, and then it was resuspended. From this re-suspended urine sample, a drop was placed in a clean glass slide using a micropipette and then covered by a coverslip. This slide preparation was then counted for a total number of pus cells under 40× magnification of a compound microscope by trained and experienced technicians (routine method). The presence of ≥5 WBCs/hpf was considered significant pyuria by this method. Microscopic hematuria was defined as presence of ≥3 red blood cell/hpf.

Using the third urine sample, TP performed a microscopic examination of uncentrifuged urine loaded in a Neubauer’s chamber under 40× magnification (chamber counting method). The number of WBCs per mm ^3^ was calculated by multiplying the total number of WBCs in the four corner squares of the chamber by a factor of 2.5. ^10^ Significant pyuria by this method was defined as ≥10 WBCs per mm ^3^. ^11^

### Exclusion of patients with ASB

Among patients without a positive UTI symptom screening, we further excluded those in whom urine culture showed significant growth of organisms that could represent asymptomatic bacteriuria. We also excluded patients with contaminated urine cultures and those in whom the treating team administered any UTI-directed antibiotics.

Workup and treatment decisions were taken by the treating medical units. We followed the patients during their hospital stay and noted their discharge diagnosis.

### Statistical analysis

The data were compiled using Microsoft Excel and analyzed using Stata/IC 12.1 for Windows (StataCorp. 2012. Stata Statistical Software: Release 12.1. College Station, TX). Categorical variables were expressed as percentages and frequencies, and continuous variables were reported as mean and standard deviation or median with the interquartile range as appropriate. The Shapiro-Wilk test was used to assess the normality of continuous variables. Categorical variables were compared using the Chi-square test. The strength of association was expressed as odds ratios with their 95% confidence intervals. A *P-value* less than 0.05 was considered statistically significant.

## Results

**Figure 1.**
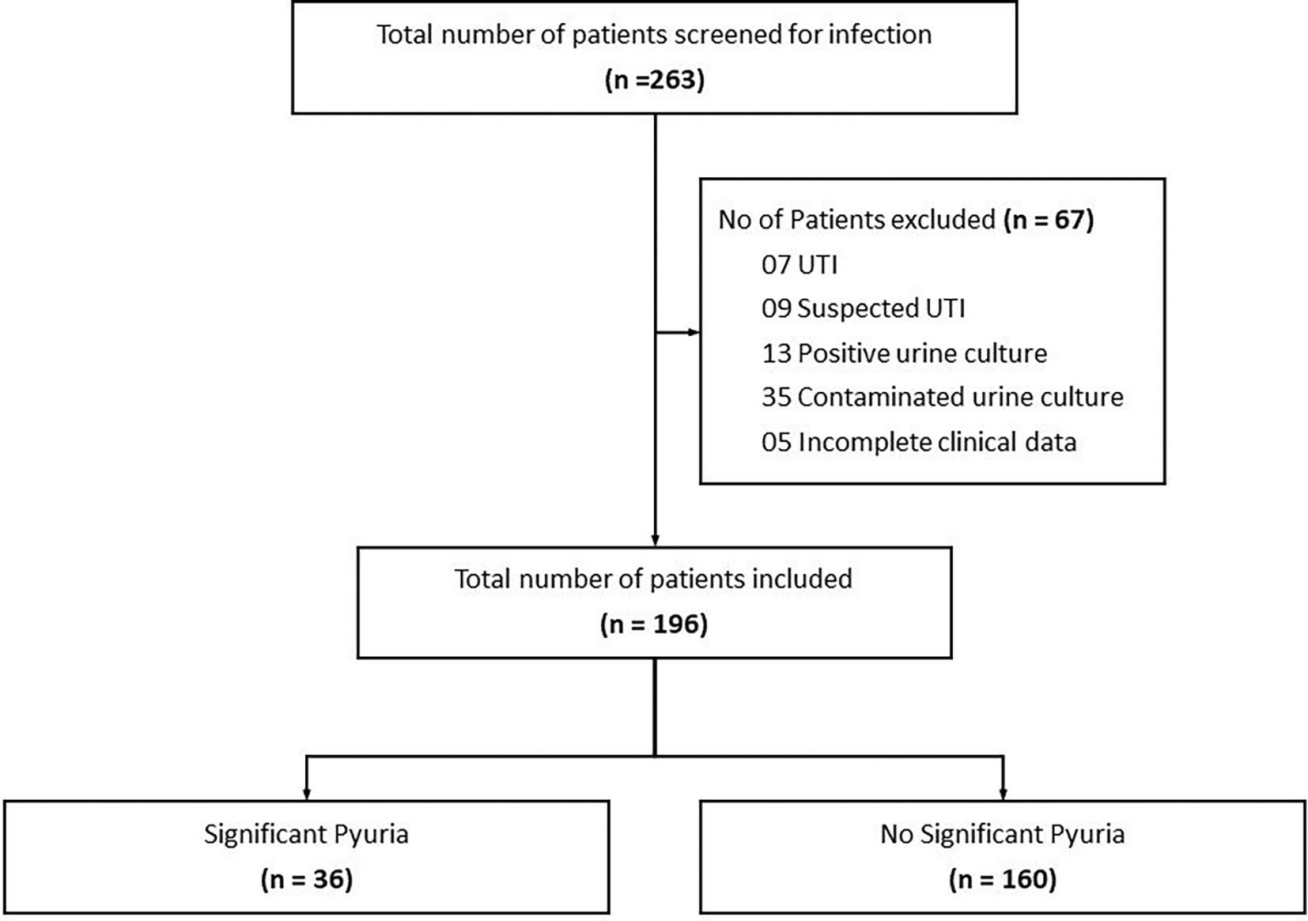
STROBE diagram depicting flow of participants through the study.

During the study period, 256 patients with suspected non-UTI diagnosis were recruited. The STROBE diagram ( Figure 1) shows the enrolment of patients in the study. Finally, 196 patients with an unambiguous diagnosis of a non-UTI condition were chosen as the study group. Of 196 patients, 113 (57.7%) were males. The median (IQR) age of the study population was 42 (29-55 years). Most (175[89.3%]) patients were hospitalized for an infection. The major diagnostic categories in the remainder (21 patients) were metabolic disorders like diabetic ketoacidosis in 5, connective tissue disorders in 4, cardiovascular diseases in 3, hepatic diseases in 2, malignancies in 2 and central nervous system, renal and gastrointestinal involvement in one patient each. The major co-morbidities in the study population were diabetes mellitus 48(24.5%), systemic hypertension 29(14.8%), chronic kidney disease 14(7.1%), coronary artery disease 10(5.1%), autoimmune diseases 10(5.1%), malignancy 8(4.1%), chronic liver disease 7(3.6%) and HIV infection 6(3.1%). The final diagnosis at discharge of these patients can be found at https://doi.org/10.6084/m9.figshare.24454228.v2.

### Comparison of the two methods of detecting pyuria

By routine method, 56 (28.3%) patients had significant pyuria. Of them, 29 (52%) had 5-10 pus cells/hpf, 19 (34%) had 11-20 pus cells/hpf, and 8 (14%) had ‘plenty’ of pus cells/hpf. By chamber counting method, 36 (18.2%) had significant pyuria. Considering the chamber counting method as the gold standard, the routine method was 69.4(63–75.8) % sensitive and 80.6(75.1–86.2) % specific. The positive and negative predictive values were 44.6 (37.7–51.6) % and 92.1 (88.4–95.9) %.

### Associations of significant pyuria

We compared the distribution of previously known and clinically intuitive risk factors for significant pyuria among patients with and without it ( Table 1). The microscopic hematuria was the only factor significantly associated with pyuria (Odds ratio 3.74[1.65–8.50]; P=0.002).

**Table 1.** Associations of significant pyuria.

Characteristic	Patients with pyuria ^a^ (N=36)	Patients without pyuria (N=160)	Unadjusted OR (95% CI)	P-value
Age (years)	41(27.5–52)	42 (29.5–55)	0.99 (0.98–1.02)	0.814
Female sex	15	68	1.03 (0.49–2.1)	0.927
Diabetes mellitus	10	38	1.24 (0.55–2.79)	0.612
Hypertension	3	26	0.47 (0.13–1.64)	0.236
Chronic kidney disease	2	12	0.73 (0.16–3.39)	0.683
Presence of fever	33	149	0.81 (0.21–3.07)	0.759
Current antibiotic use	26	126	0.70 (0.31–1.60)	0.398
Total leucocyte count (cells/mm ^3^)	10030 (8130–14200)	10455(6050–14462.5)	1.00 (0.99–1.00)	0.689
Serum creatinine (mg/dL)	1.15(0.75–2.2)	0.9(0.69–1.3)	1.03 (0.91–1.16)	0.644
Acute kidney injury	11	31	1.77 (0.79–3.99)	0.166
Dipstick glucosuria	8	23	1.70 (0.69–4.19)	0.244
Dipstick proteinuria	22	76	1.73(0.83–3.63)	0.140
Microscopic hematuria	13	21	3.74 (1.65–8.50)	0.002

Data are represented as frequency (N) or median (IQR).

^a^ Significant pyuria by chamber counting method.

## Discussion

In this prospective study including hospitalized general medical patients with a non-UTI diagnosis, we observed that about 1 out of 5 patients had significant pyuria. Further, microscopic hematuria was observed to be significantly associated with pyuria. We did not observe any association with pyuria and female sex, diabetes mellitus, hypertension, AKI, and infections.

In the study done by Hooker et al., including hospitalized patients with infections outside the urinary tract, nearly one-third of adults (43 of 144; 29.9%) patients had pyuria (≥5 WBCs/hpf). ^7^ They found that pyuria was more common in women and patients with genital tract infections. In their study, urine culture was positive in 42% of adult patients, and 38% were reclassified as UTI. We excluded patients with symptoms and signs of UTI, those with a positive urine culture, and also those patients who received UTI-directed antibiotics. Hence, we did not reclassify anyone as UTI. Moreover, we have included patients with non-infectious diseases also. Using the routine method, we found 28% had significant pyuria of >5 cells/hpf. However, using the chamber counting method, the proportion was noted to be 18%.

Pyuria expressed as pus cells/mm ^3^ using unspun urine is a more accurate and reproducible method than counting the number of pus cells per hpf in using centrifuged urine. ^12^^,^^13^ We found that the diagnostic performance characteristics of the routine method is modest in comparison to chamber counting method with a sensitivity of 70% and specificity of 81%. As early as 1962, Stansfeld had reported that the co-efficient of variation of the chamber counting method is 8.8%, while it is 21% for the routine method. ^10^ In the context of UTI diagnosis, studies have shown that more than 96% of symptomatic UTI with significant bacteriuria had ≥10 cells/mm ^3^, while this finding was present in less than one percent of asymptomatic non-bacteriuric patients. ^14^ However, detecting pyuria using the chamber counting method could be time-consuming and requires expertise. Moreover, operators should be cognizant of possible calibration and observer errors while using a hemocytometer. ^15^

It is important to note that 18% of hospitalized patients had significant pyuria in our study. The cause of pyuria in this population is neither UTI nor ASB. Microscopic hematuria was found to be strongly associated with pyuria, and thus, we can infer that microscopic urinary abnormalities are not uncommon in this population. In a recent review on sterile pyuria, gynaecological infections, urethritis, balanitis, viral infections of the genitourinary tract, prostatitis, genitourinary tuberculosis, and current use of antibiotics have been listed as causes related to infection for this finding. ^11^ The causes unrelated to infection are urinary catheter use, urological procedures, urinary tract stones or neoplasms, papillary necrosis, inflammatory diseases like SLE, etc. Most of these conditions were absent in our study population. However, it is possible that asymptomatic infections of urinary tract and/or urological abnormalities like clinically silent renal stones could have been present in a proportion of them. Of note, previous studies have found that pyuria is more common with female sex and diabetes. ^7^^,^^16^ We did not find these associations in our study population, but this could possibly because the study was underpowered to a significant difference.

Finding pyuria might also trigger the ordering of urine cultures even when patients have no clinical features suggestive of UTI. A positive urine culture in such a setting most likely represents ASB, a condition that accounts for 20-45% of positive urine cultures in hospitalized patients. ^6^^,^^17^ Inappropriate use of antibiotics for ASB is quite common in hospital settings, and the presence of concomitant pyuria is an important driver for this. ^18^^,^^19^ Thus, un-indicated urinalysis should be avoided in hospitalized patients.

Our study has the merit that pyuria was ascertained using the chamber counting method. We found that using a cut-off of ≥5 WBCs/hpf would lead to overdiagnosis of pyuria in about 10% more patients. However, our study would have been more informative had we checked for persistent pyuria and other urinary casts.

In conclusion, we found that abnormal microscopic urinalysis findings like pyuria and hematuria can be present in a proportion of hospitalized patients across a wide range of diagnostic categories. Estimating the number of pus cells per high power field in centrifuged urine has only modest diagnostic performance compared to the chamber counting method. Urinalysis may be best used as a ‘rule-out’ test for UTI rather than a specific test for diagnosing UTI. Unnecessary ordering of urinalysis should be avoided, and the presence of pyuria has to be interpreted in the light of the given clinical context. Finally, a positive urinalysis in a patient without typical symptoms of UTI should not reflexively prompt urine culture testing and/or antibiotic treatment. Clinical judgement is warranted.

### Ethics and consent

The study protocol was reviewed and approved by the Institute Ethics Committee (Human Studies), JIPMER, Puducherry (No. JIP/IEC/2020/202; dated April 19, 2021).

Written informed consent was obtained from all study participants.

## Author contributions

Study design: Surendran Deepanjali, Jharna Mandal

Data acquisition: Praveenkumar Thyagaraju, Hariswar Pari Thenmozhi

Data analysis: Surendran Deepanjali, Hariswar Pari Thenmozhi

Drafting of manuscript: Surendran Deepanjali, Hariswar Pari Thenmozhi, Praveenkumar Thyagaraju

Critical revision of the manuscript: Jharna Mandal

## Data Availability

Figshare: Sterile pyuria in hospitalized general medical patients, DOI https://doi.org/10.6084/m9.figshare.24454228.v2. ^20^ Data are available under the terms of the Creative Commons Attribution 4.0 International license (CC-BY 4.0).
