# Supplementary material for: Pyuria in hospitalized general medical patients without urinary tract infection
Source: F1000Res. 2024 May 22;13:291. Originally published 2024 Apr 18. [Version 2] doi: 10.12688/f1000research.144853.2 (PMC11439123; doi:10.12688/f1000research.144853.2)
Supplement: Peer review history

# Reviewer report: Reviewer response for version 2

**DOI:** 10.5256/f1000research.166585.r309828

**Published:** 2024-09-28

**Authors:** James Kojo Prah

**Affiliations:** 1 University of Cape Coast, Cape Coast, Central, Ghana

This study investigating the presence of asymptomatic pyuria in hospitalized patients has been well written, and the methods well described.

 Some recommendations:

 1. The problem that necessitated the conduct of this study needs to be clarified, especially in the study population.

 2. Since the study compared chamber counting and routine methods, the results (sensitivity, specificity, etc.) must be discussed, emphasizing the implications for clinical practice. The manuscript did not include such discussions.

 3. Did your search of available literature reveal any similar studies that also compared these two methods? If so, could you include these in the discussions? If no, clearly indicate this as well

Is the work clearly and accurately presented and does it cite the current literature?

Yes

If applicable, is the statistical analysis and its interpretation appropriate?

Yes

Are all the source data underlying the results available to ensure full reproducibility?

Yes

Is the study design appropriate and is the work technically sound?

Yes

Are the conclusions drawn adequately supported by the results?

Yes

Are sufficient details of methods and analysis provided to allow replication by others?

Yes

Reviewer Expertise:

Occupational health, parasitology, public health

I confirm that I have read this submission and believe that I have an appropriate level of expertise to confirm that it is of an acceptable scientific standard.

## References

[1] : Pyuria in hospitalized general medical patients without urinary tract infection. *F1000Research* .2024;13: 10.12688/f1000research.144853.2 10.12688/f1000research.144853.2 PMC1143912339346951

# Reviewer report: Reviewer response for version 2

**DOI:** 10.5256/f1000research.166585.r309826

**Published:** 2024-08-22

**Authors:** Alan J Wolfe

**Affiliations:** 1 Microbiology and Immunology, Loyola University Chicago, Chicago, Illinois, USA

In this submission, the authors investigate pyuria in hospitalized patients without a UTI diagnosis. If the authors wish to perpetuate old dogma, then this contribution is just fine BUT if they wish to be part of the future then this study is totally useless and in fact could be detrimental as it perpetuates old assumptions and habits.

 For starters, the authors do not even mention of the urobiome, the resident community of microbes of the urinary tract. The fact that the article does not even mention the urobiome - discovered more than a decade ago - is a serious oversight. These authors have studied the past with little relevance for the future. How can the authors completely ignore the last 14 years of research? Are they completely unaware of this transformational research or have they chosen to ignore it? If the former, they might start educating themselves by reading the 2023 article by Brubaker and others entitled “Tarnished gold” (Brubaker L, et. al., 2023 [Ref 1]) and then diving into many of the cited papers.

 Because the adult urinary tract is not sterile, several terms must be re-evaluated, redefined, and in some cases jettisoned. For example, “asymptomatic bacteriuria” is an anachronism. What does this term mean when almost everyone is colonized by microbes including those without symptoms. The current definition focuses on the detection of the usual suspects, usually E. coli, by the standard urine culture (SUC) method used by most clinical microbiology laboratories around the world. While E. coli is the most commonly detected bacterial species in individuals with UTI symptoms, it is only detected in ~40-50% of individuals with symptoms, not the usually reported 70-90%. The latter range is due to skewing by the use of the SUC, which was designed to detect E. coli. As argued in “Tarnished Gold,” SUC no longer should be considered the "gold standard" because it is unable to detect fastidious emerging uropathogens species, such as Aerococcus, Actinotignum, and Streptococcus anginosus, which are extremely common in individuals with lower urinary tract symptoms. It even underreports some typical uropathogens, especially Enterococcus (for example, see Hochstedler BR, et. al., 2022 [Ref 2]). Thus, the authors exclusion of individuals with SUC-positive results biases their study.

 Now, let’s talk about the term “sterile pyuria.” Given that SUC misses very common fastidious uropathogens, the pyuria noted in the SUC-negative individuals likely indicates the presence of undetected uropathogens. Again, read Brubaker et al., 2023 for a start. Since very few adult females have a sterile urinary tract, then if pyuria is present, clinicians should consider the possibility that there is quite possibly a microbiological cause. Why clinicians, clinical microbiologists, and researchers in the past didn't question the finding of sterile pyuria and begin to suspect the SUC method to be inadequate is beyond me. The presence of immune cells (WBCs) and/or red blood cells (RBCs) should have immediately suggested the possibility of an infectious source that was undetected.

 Finally, there is convincing evidence that urines must be read quickly to obtain accurate WBC counts, something that often does not happen in a busy clinic/clinical micro lab. This is because WBCs die relatively rapidly. To begin to learn about this phenomenon, the authors might read Brubaker L, et al., 2023 (Ref 3).

 In summary, this article is at least a decade out of date. Readers should be made aware of the new discoveries and begin to incorporate them into their thinking, for the sake of their patients.

Is the work clearly and accurately presented and does it cite the current literature?

No

If applicable, is the statistical analysis and its interpretation appropriate?

Yes

Are all the source data underlying the results available to ensure full reproducibility?

Yes

Is the study design appropriate and is the work technically sound?

No

Are the conclusions drawn adequately supported by the results?

Yes

Are sufficient details of methods and analysis provided to allow replication by others?

Yes

Reviewer Expertise:

urinary microbiome

I confirm that I have read this submission and believe that I have an appropriate level of expertise to state that I do not consider it to be of an acceptable scientific standard, for reasons outlined above.

## References

[1] : Tarnished gold—the “standard” urine culture: reassessing the characteristics of a criterion standard for detecting urinary microbes. *Frontiers in Urology* .2023;3: 10.3389/fruro.2023.1206046 10.3389/fruro.2023.1206046 PMC1232731640778073

[2] : Urinary microbiota of women with recurrent urinary tract infection: collection and culture methods. *Int Urogynecol J* .2022;33(3) : 10.1007/s00192-021-04780-4 563-570 10.1007/s00192-021-04780-4 33852041 PMC8514570

[3] : Microbiologist in the clinic: coitally related symptoms with negative urine cultures. *Int Urogynecol J* .2023;34(11) : 10.1007/s00192-023-05662-7 2701-2704 10.1007/s00192-023-05662-7 37837460

# Reviewer report: Reviewer response for version 1

**DOI:** 10.5256/f1000research.158706.r273342

**Published:** 2024-05-13

**Authors:** Kartikeya Cherabuddi

**Affiliations:** 1 University of Florida, Gainesville, FL, USA

This is an important study and article detailing the high presence of asymptomatic pyuria in a hospitalised population. The authors have done a superb job in detailing the introduction, methods, results and discussion. 

 Some recommendation to further enhance the article:

 1. Please list a reference for this statement: using the chamber counting method, which is a more accurate method of quantifying pyuria. Please include a newer reference than #8 if available. 

 2. We found that using a cut-off of ≥5 WBCs/hpf would lead to overdiagnosis of pyuria in about 10% more patients. Would a cut-off of >= 10 WBCs/hpf in the routine method be comparable to the chamber counting method? 

 3. Consider modification to: Unnecessary and routine ordering of urinalysis should be avoided, and the presence of pyuria has to be interpreted in the light of the given clinical context. Finally, a positive urinalysis with pyuria in an asymptomatic patient should not prompt urine culture testing and/or antibiotic treatment. 

 4. Consider listing the limitations of the chamber counting method - time, need for training/ expertise etc as relevant. 

 5. Were patients with recent urinary catheterisation excluded? If so, please state it. 

 5. Minor typographical errors 

 a) had ‘plenty’ of us cells/hpf.

 b) medicine side lab

Is the work clearly and accurately presented and does it cite the current literature?

Yes

If applicable, is the statistical analysis and its interpretation appropriate?

Yes

Are all the source data underlying the results available to ensure full reproducibility?

Partly

Is the study design appropriate and is the work technically sound?

Yes

Are the conclusions drawn adequately supported by the results?

Yes

Are sufficient details of methods and analysis provided to allow replication by others?

Yes

Reviewer Expertise:

Infectious diseases

I confirm that I have read this submission and believe that I have an appropriate level of expertise to confirm that it is of an acceptable scientific standard.

# Response

**Published:** 2024-05-14

**Authors:** Surendran Deepanjali

**Affiliations:** Medicine, Jawaharlal Institute of Postgraduate Medical Education and Research (JIPMER), Dhanvantri Nagar, Puducherry, India

**Response to reviewer**

**Comment**

 This is an important study and article detailing the high presence of asymptomatic pyuria in a hospitalised population. The authors have done a superb job in detailing the introduction, methods, results and discussion.

**Response**

 We are very grateful to the reviewer for his very kind words of appreciation.

**Comment**

 1. Please list a reference for this statement: using the chamber counting method, which is a more accurate method of quantifying pyuria. Please include a newer reference than #8 if available.

**Response**

 We did a repeat literature search whether any more recent studies comparing the two methods of pyuria have been published, but could not find any. However, we found a study more recent than reference 8 which describes that chamber counting method is more accurate and hence we will include this reference in the modified version of the manuscript. (Saito A, Kawada Y. Reliability of pyuria detection method. Infection. 1994;22 Suppl 1:S36-7.)

**Comment**

 2. We found that using a cut-off of ≥5 WBCs/hpf would lead to overdiagnosis of pyuria in about 10% more patients. Would a cut-off of >= 10 WBCs/hpf in the routine method be comparable to the chamber counting method?

 The reviewer is correct in postulating that a cut-off of ≥ 10 cells/hpf would be more comparable to the chamber counting method. Such a cut-off would be more specific and have a better positive predictive value. We could have done this analysis using our dataset; however, our lab reports the pus cells/hpf as 5-10 and 11-20 etc, hence it is not possible to find out how many had exactly 10 WBCs/hpf. We came across this comparison (between 5 versus 10 WBCs/hpf) in the study quoted in the previous response, where Saito et al observed a sensitivity of 52% and a specificity of 86% for ≥5 WBCs/hpf versus a sensitivity of 48% and 87% for ≥10 WBCs/hpf. Nevertheless, we would like to bring to the kind attention of the reviewer that presence of ≥5 WBCs/hpf in urine has been reiterated as an entry criterion for UTI in a very recently published consensus definition for UTI (Bilsen MP, Conroy SP, Schneeberger C, et al.; UTI Reference Standard Consensus Group. A reference standard for urinary tract infection research: a multidisciplinary Delphi consensus study. Lancet Infect Dis. 2024 Mar 5:S1473-3099(23)00778-8.)

**Comment**

 3. Consider modification to: Unnecessary and routine ordering of urinalysis should be avoided, and the presence of pyuria has to be interpreted in the light of the given clinical context. Finally, a positive urinalysis with pyuria in an asymptomatic patient should not prompt urine culture testing and/or antibiotic treatment.

 We will be happy to make this change in the Conclusion section.

**Comment**

 4. Consider listing the limitations of the chamber counting method - time, need for training/ expertise etc as relevant.

**Response**

 In the Discussion section of the manuscript (Paragraph 3) we plan to include the following statements ***: “However, detecting pyuria using the chamber counting method could be time -consuming and requires expertise. Moreover, operators should be cognizant of possible calibration and observer errors while using a hemocytometer.”*** (Gadeholt H. Counting of cells in urine. The variability of haemocytometer counts. Acta Med Scand. 1968 Jan-Feb;183(1-2):9-16.)

**Comment**

 5. Were patients with recent urinary catheterisation excluded? If so, please state it.

**Response**

 Yes, they were excluded. We will state it in exclusion criteria.

**Comment**

 5. Minor typographical errors

 a) had ‘plenty’ of us cells/hpf.

 b) medicine side lab

**Response**

 We thank the reviewer for pointing them out. The lab in our department is actually called a ‘side lab’, but we will change this to ‘medicine lab’ to avoid confusion.

 We thank the reviewer again for his constructive comments.

# Reviewer report: Reviewer response for version 1

**DOI:** 10.5256/f1000research.158706.r273337

**Published:** 2024-05-10

**Authors:** Rani Diana Sahni

**Affiliations:** 1 Christian Medical College Vellore Association, Vellore, Tamil Nadu, India

Dear authors, 

 Thank you for submitting your work for review. 

 In our daily work we come across protocols that are sometimes questionable but have not been revised due to a lack of sufficient evidence to change the norm. This topic is one such issue. 

**Please find below the comments / suggestions of this review.**

**1. Introduction:**

 "Urinalysis for detecting pyuria is a commonly ordered test in hospitalized patients. It is considered one of the ‘routine’ or ‘default’ investigations after hospital admission..........More recent studies show that urinalysis is often ordered without proper indication in the emergency department." 

 The opening statement in the introductory section and the last sentence in the same paragraph, suggests that these tests are over ordered and without a reasonable clinical basis. 

 However, urine microscopy to detect "pyuria" has its merits and is ordered as a cheaper and quicker method to "rule out" a urinary tract pathology rather than to "rule-in" an infective or non-infective disease in an emergency department and can be used as the only test in a resource limited setting.

 Therefore, the introductory passage needs to be modified. 

**2. Methodology:**

 1. The study has been carried out among hospitalized patients; however, this test is more relevant in an out-patient/emergency setting. Please explain why the hospitalized patient subset was chosen for this study.

 2. Pus cells / WBC can be produced in other non-infective and inflammatory clinical settings and therefore does not always indicate an infection (UTI). 

 Therefore, the reasons for hospitalization, need to be addressed. Please include the same.

 It is relevant as they could contribute to the presence of inflammatory cells in urine. 

 To name a few: Renal pathology inclusive of calculi and tumors, BPH, Bacterial vaginosis, urethritis. 

 As these were all asymptomatic participants, the reason most likely to be associated with pyuria may then be determined. 

 3. Pus cells may also be seen in patients with a recent history of UTI / recent catheter removals or urosepsis without a lower UTI presentation. The exclusion criteria should include: patients who have been on antibiotics in the last two weeks / history of UTI in the last 2-4 weeks / urosepsis. 

 Please explain if these were a part of the exclusion criteria.

**Concluding remark: **The ultimate sample size is small to describe the differences between two microscopic methods and reasons for pyuria. 

 However, The most relevant information in the manuscript seems to be the comparison between the 2 microscopic methods which may be highlighted with a description of the clinical cases that may benefit from a urine microscopy test.

 Best wishes.

Is the work clearly and accurately presented and does it cite the current literature?

Yes

If applicable, is the statistical analysis and its interpretation appropriate?

Yes

Are all the source data underlying the results available to ensure full reproducibility?

Yes

Is the study design appropriate and is the work technically sound?

Partly

Are the conclusions drawn adequately supported by the results?

Yes

Are sufficient details of methods and analysis provided to allow replication by others?

No

Reviewer Expertise:

Genitourinary infection diagnostics, antimicrobial susceptibility

I confirm that I have read this submission and believe that I have an appropriate level of expertise to confirm that it is of an acceptable scientific standard, however I have significant reservations, as outlined above.

# Response

**Published:** 2024-05-13

**Authors:** Surendran Deepanjali

**Affiliations:** Medicine, Jawaharlal Institute of Postgraduate Medical Education and Research (JIPMER), Dhanvantri Nagar, Puducherry, India

**Response to reviewer**

 We are grateful to the reviewer for the critical appraisal of our manuscript and for providing valuable feedback. We hope to address the concerns raised by the reviewer as much as possible and make modifications in the revised version of our manuscript which will be uploaded after obtaining further comments from more reviewers (as advised by the editorial team). Please find the point-to-point responses for your comments below. Please note that the parts planned to be modified are italicized and bolded for ready reference.

**Comment**

**1. Introduction:**

 "Urinalysis for detecting pyuria is a commonly ordered test in hospitalized patients. It is considered one of the ‘routine’ or ‘default’ investigations after hospital admission..........More recent studies show that urinalysis is often ordered without proper indication in the emergency department." 

 The opening statement in the introductory section and the last sentence in the same paragraph, suggests that these tests are over ordered and without a reasonable clinical basis. 

 However, urine microscopy to detect "pyuria" has its merits and is ordered as a cheaper and quicker method to "rule out" a urinary tract pathology rather than to "rule-in" an infective or non-infective disease in an emergency department and can be used as the only test in a resource limited setting.

 Therefore, the introductory passage needs to be modified. 

**Response**

 We agree with the reviewer that urinalysis is a cheap and quick ‘rule-out’ test in the ED (except in those with obstructive uropathy or absolute neutropenia). To that effect, we will modify the Introduction as given below. We will also add a new reference to point this out.

 The second paragraph of Introduction:

 “In patients with classic clinical symptoms and signs of UTI, the presence of pyuria and significant bacteriuria helps in ascertaining the diagnosis of UTI. ***Also, it has been observed that urinalysis serves as a useful “rule-out” test for UTI*** ***(Advani SD, North R, Turner NA, Ahmadi S, Denniss J, Francis A, Johnson R, Hasan A, Mirza F, Pardue S, Rao M, Rosshandler Y, Tang H, Schmader KE, Anderson DJ. Performance of Urinalysis Parameters in Predicting Urinary Tract Infection: Does One Size Fit all? Clin Infect Dis. 2024 Apr 26:ciae230.) *** However, in those without typical clinical features of UTI, the presence of pus cells in urine detected by a routinely done microscopy examination often leads to diagnostic confusion. This is especially so if pyuria is accompanied by bacteriuria, which is detected by a urine culture. This condition, which was proposed to be called ‘Bacteriuria/Pyuria of Clinically Undetermined Significance (BPCUS),’ is indeed not so uncommon in hospital settings. ^4^”

**Comment**

**2. Methodology:**

 1. The study has been carried out among hospitalized patients; however, this test is more relevant in an out-patient/emergency setting. Please explain why the hospitalized patient subset was chosen for this study.

 3. Pus cells may also be seen in patients with a recent history of UTI / recent catheter removals or urosepsis without a lower UTI presentation. The exclusion criteria should include: patients who have been on antibiotics in the last two weeks / history of UTI in the last 2-4 weeks / urosepsis. 

 Please explain if these were a part of the exclusion criteria.

**Response**

 We thank the reviewer for pointing this out. All patients were recruited from the emergency department setting within 24 hours of presentation. We confirm that we have excluded patients on urinary catheter (already mentioned as an exclusion criterion) and those with urosepsis (excluded all patients with suspected/confirmed urosepsis). Further, we did exclude patients who have been hospitalized or treated with antibiotics in the previous 2 weeks of admission to our hospital. Please find the modifications effected below.

 Methods

 We did a prospective observational study between April 2021 to June 2022 among adult patients presenting to casualty and who were subsequently admitted under the Department of Medicine, JIPMER. The study protocol was reviewed and approved by the Institute Ethics Committee (Human Studies), JIPMER, Puducherry (No. JIP/IEC/2020/202; dated April 19, 2021). Written informed consent was obtained from all study participants and the study was conducted in compliance with the Declaration of Helsinki. Our study population included adult patients hospitalized with a non-UTI diagnosis. **We excluded patients who had a positive UTI symptom screening at admission as well as those on indwelling urinary catheter. *We also excluded who had a recent (past 2 weeks) hospitalization or exposure to antibiotics.*** ***Patients were recruited within 24 hours of hospitalization.*** We defined positive UTI as the presence of at least two or more typical symptoms or signs suggestive of UTI like dysuria, urinary frequency, urgency, hesitancy, lower abdominal pain, loin pain, suprapubic tenderness, and renal angle tenderness.

**Comment**

 2. Pus cells / WBC can be produced in other non-infective and inflammatory clinical settings and therefore does not always indicate an infection (UTI). 

 Therefore, the reasons for hospitalization, need to be addressed. Please include the same.

 It is relevant as they could contribute to the presence of inflammatory cells in urine. 

 To name a few: Renal pathology inclusive of calculi and tumors, BPH, Bacterial vaginosis, urethritis. 

 As these were all asymptomatic participants, the reason most likely to be associated with pyuria may then be determined.

**Response**

 The reviewer is correct in pointing out that pus cells in urine is not always indicative of UTI and that non-infective and inflammatory conditions can cause it. Our study shows that infections other than UTI also could also result in urine pus cells. We would like to bring to the kind attention of the reviewer that we do discuss regarding other possible causes of pyuria like urethritis and renal calculi in the Discussion section of the manuscript, which we are quoting here, “In a recent review on sterile pyuria, gynaecological infections, urethritis, balanitis, viral infections of the genitourinary tract, prostatitis, genitourinary tuberculosis, and current use of antibiotics have been listed as causes related to infection for this finding. ^9^ The causes unrelated to infection are urinary catheter use, urological procedures, urinary tract stones or neoplasms, papillary necrosis, inflammatory diseases like SLE, etc. **Our study population did not include those with these diagnoses. However, it is possible that asymptomatic infections of urinary tract and/or urological abnormalities like clinically silent renal stones could have been present in a proportion of them.** “  

 The presenting complaints at admission and the discharge diagnosis of all the study participants are available in the open access dataset attached with the manuscript. It was not possible to tabulate all diagnoses to be included in the manuscript. However, we will include the major diagnostic categories for all 196 participants the Results section as follows: **“** **Most (175[89.3%]) patients were hospitalized for an infection. *The major diagnostic categories in the remainder (21 patients) were metabolic disorders like diabetic ketoacidosis in 5,* *connective tissue disorders in 4, cardiovascular diseases in 3, hepatic diseases in 2, malignancies in 2 and central nervous system, renal and gastrointestinal involvement in one patient each.”***

**Concluding remark: **The ultimate sample size is small to describe the differences between two microscopic methods and reasons for pyuria. 

 However, The most relevant information in the manuscript seems to be the comparison between the 2 microscopic methods which may be highlighted with a description of the clinical cases that may benefit from a urine microscopy test.

**Response**

 The primary objective of the study was to see the frequency of pyuria in patients with a non-UTI diagnosis and the sample size may not have been adequate to test the diagnostic performance characteristics of the ‘routine’ method. We do discuss about the merits of chamber counting method for pyuria detection in Paragraph 3 of Discussion section. However, as the reviewer suggested we will include this point in Conclusion section. Further, we will give a suggestion regarding the utility of this test as a ‘rule-out’ rather than a ‘rule-in’ test for UTI. Please read the modified Conclusion here:

 “In conclusion, we found that abnormal microscopic urinalysis findings like pyuria and hematuria can be present in a proportion of hospitalized patients across a wide range of diagnostic categories. ***Estimating the number of pus cells per high power field in centrifuged urine has only modest diagnostic performance compared to the chamber counting method. Urinalysis may be best used as a ‘rule-out’ test for UTI rather than a specific test for diagnosing UTI. *** Unnecessary ordering of urinalysis should be avoided, and the presence of pyuria has to be interpreted in the light of the given clinical context.

**Comment**

- **Is the study design appropriate and is the work technically sound?**

Partly

- **Are sufficient details of methods and analysis provided to allow replication by others?**

No

**Response**

 We believe we have explained the methods and analysis in detail; however, we will be happy to clarify them further if deemed necessary by the reviewer.

 Thank you once again for reviewing our study and providing constructive comments!!
